# Do We Really Need Hazard Prevention at the Expense of Safeguarding Death Dignity in COVID-19?

**DOI:** 10.3390/diagnostics11101913

**Published:** 2021-10-15

**Authors:** Cristoforo Pomara, Francesco Sessa, Domenico Galante, Lorenzo Pace, Antonio Fasanella, Nunzio Di Nunno, Massimiliano Esposito, Monica Salerno

**Affiliations:** 1 Department of Medical, Surgical and Advanced Technologies “G.F. Ingrassia”, University of Catania, 95121 Catania, Italy; massimiliano.esposito91@gmail.com (M.E.); monica.salerno@unict.it (M.S.); 2 Department of Clinical and Experimental Medicine, University of Foggia, 71122 Foggia, Italy; francesco.sessa@unifg.it; 3 Istituto Zooprofilattico Sperimentale della Puglia e della Basilicata, 71121 Foggia, Italy; domenico.galante@izspb.it (D.G.); lorenzo.pace@izspb.it (L.P.); antonio.fasanella@izspb.it (A.F.); 4 Department of History, Society and Studies on Humanity, University of Salento, 73100 Lecce, Italy; nunzio.dinunno@unisalento.it

**Keywords:** autopsy, COVID-19, RT-PCR, SARS-CoV-2, viral transmission

## Abstract

To date, little is known regarding the transmission risks of SARS-CoV-2 infection for subjects involved in handling, transporting, and examining deceased persons with known or suspected COVID-19 positivity at the time of death. This experimental study aims to define if and/or how long SARS-CoV-2 persists with replication capacity in the tissues of individuals who died with/from COVID-19, thereby generating infectious hazards. Sixteen patients who died with/from COVID-19 who underwent autopsy between April 2020 and April 2021 were included in this study. Based on PMI, all samples were subdivided into two groups: ‘short PMI’ group (eight subjects who were autopsied between 12 to 72 h after death); ‘long PMI’ (eight subjects who were autopsied between 24 to 78 days after death). All patients tested positive for RT-PCR at nasopharyngeal swab both before death and on samples collected during post-mortem investigation. Moreover, a lung specimen was collected and frozen at −80 °C in order to perform viral culture. The result was defined based on the cytopathic effect (subjective reading) combined with the positivity of the RT-PCR test (objective reading) in the supernatant. Only in one sample (PMI 12 h), virus vitality was demonstrated. This study, supported by a literature review, suggests that the risk of cadaveric infection in cases of a person who died from/with COVID-19 is extremely low in the first hours after death, becoming null after 12 h after death, confirming the World Health Organization (WHO) assumed in March 2020 and suggesting that the corpse of a subject who died from/with COVID-19 should be generally considered not infectious.

## 1. Introduction

Since the onset of the 2019 Coronavirus Pandemic (COVID-19), the scientific community has been working to produce guidelines and recommendations to help healthcare professionals proceed safely with disease management and investigation. Most of the published articles have addressed multiple aspects, clarifying clinical presentation [1,2,3], diagnostic tests [4,5,6], treatment modalities [7,8,9], and hospitalization management [10,11,12]. Pathological and laboratory issues, including autopsy procedures and cadaver handling, have not yet been well characterized. Several governments imposed severe restrictions concerning the corpse management of subjects who died with/from COVID-19, raising important questions, especially from an ethical perspective [13,14]. These restrictions were imposed without any individualized risk assessment: they were adopted in a prudent way in order to reduce the infectious hazards [15,16].

For example, in Italy, the first European country to be affected by the COVID-19 pandemic, burial procedures were immediately banned on the basis of the first studies published on the environmental stability of the virus. In the first phase, the cremation was encouraged for COVID-19 deaths. Visitation of the body and funeral ceremonies were also banned, and any prayers at the closing of the coffin were suspended. Moreover, burial of the corpse with both personal items and clothes was not allowed. To minimize delays between the time of death and cremation, the deceased was taken directly to the cemetery where a brief burial rite was performed [17]. Similar recommendations were pointed out by the Autopsy Work Group of the Spanish Society of Anatomical Pathology [18] and by the Royal Collage of Pathologist [19] that discouraged to perform autopsies in positive COVID-19 cases. These indications were more restrictive compared to the guidance of World Health Organization (WHO) which in its document has suggested that with the exception of the cases of hemorrhagic fevers and cholera, the corpse of a subject who died from/with COVID-19 should be generally considered not infectious. In the same document, WHO has recommended attention in the management of the lungs during the autopsy [20]. On the contrary, the restriction of autopsies during the COVID-19 pandemic has slowed down the acquisition of data about the new virus. Early autopsies of deceased patients confirmed that SARS-CoV-2 does not just cause respiratory disease, but can also attack other vital organs, stressing the important concept to “learning from death” [21,22].

Although several studies have been performed to clarify different important aspects about the risks of SARS-CoV-2 infection for subjects involved in handling, transporting, and examining deceased persons with COVID-19 [23,24,25,26], to the best of our knowledge, no studies have been performed to investigate virus vitality in post-mortem samples.

In light of these considerations, this experimental study aims to define if and/or how long SARS-CoV-2 persists with replication capacity in the tissues of individuals who died with/from COVID-19, thereby generating a real risk of infection.

## 2. Materials and Methods

### 2.1. Patients, Autopsies and Sample Collection

**Figure 1:**
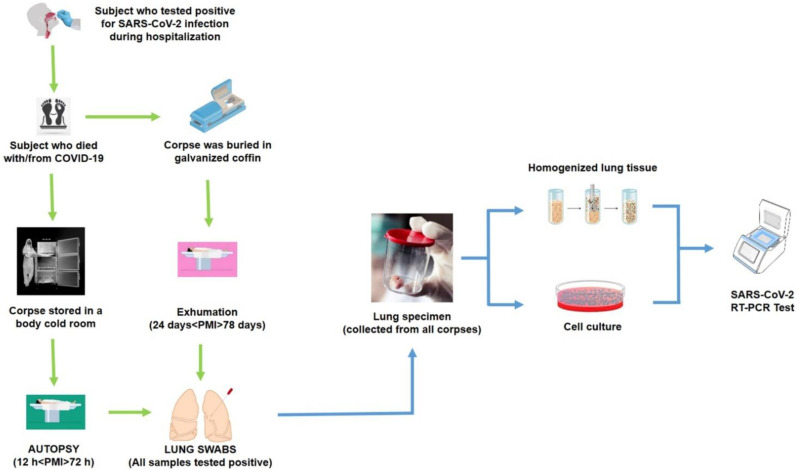
The protocol study: all samples were obtained by subjects died from/with COVID-19.

Of a total of 35 autopsies performed in our institution from April 2020 to April 2021, we selected 16 clinical and forensic autopsies of COVID-19 patients that satisfied the inclusion criteria: positivity to RT-PCR test for SARS-CoV-2 infection at the admission; positivity to RT-PCR test for SARS-CoV-2 infection at the time of death; positivity to RT-PCR test for SARS-CoV-2 infection for lung tissue sampled during autopsy. The protocol study was summarized in Figure 1.

Autopsies performed with a different post-mortem interval (PMI) were selected; based on PMI parameters, all samples were split into two groups: 8 samples constituted the short PMI group (12 h ≤ PMI ≤ 72 h), meaning that the autopsies were performed within 72 h, and 8 samples for the long PMI group (24 days ≤ PMI ≤ 78 days), meaning that the autopsies were performed on exhumed corpses after the indicated PMI; during this period, they were buried in galvanized coffins. Autopsies were conducted following international guidelines [13,20,22,27]. All autopsies were conducted according to the Letulle method [28]. Before death, all nasopharyngeal swabs collected from the subjects enrolled in the present study tested positive at the COVID-19 rRT-PCR assay [24]. The cause of death for each subject is reported in Table 1. During the autopsy, a lung swab sample was collected for each subject, confirming a positive result. Prior to fixation, three tissue fragments from the right lung and two from the left lung were collected and immediately transferred to sterile vials containing RNA Later (Cat. 76104, RNA Protect Tissue Reagent, Qiagen) and stored at −80 °C pending extraction. All tissues were sent to a biosafety level 3 (BSL-3) laboratory for viral culture.

### 2.2. Virus Isolation

For SARS-CoV-2 isolation, the Vero E6 cell line (African green monkey kidney cells) was used [29]. Cells were cultured in Eagle’s minimal essential medium (EMEM) (Life Technologies, Carisbad, CA, USA) supplemented with 10% (*v*/*v*) fetal bovine serum (FBS) (Life Technologies, Carisbad, CA, USA), and 100 U/mL penicillin and streptomycin (Life Technologies, Carisbad, CA, USA).

For the virus isolation from lung tissues, cells were plated into 25 cm^2^ cell culture flasks (Corning, New York, NY, USA) at a confluence of 70–80% in 6 mL EMEM with 10% FBS and incubated overnight at 37 °C.

The following day, lung samples were mechanically homogenized by TissueRuptor II (Qiagen, Hilden, Germany) in 3 mL of PBS. Each sample was centrifugated at 5000× *g* for 5 min and the supernatant was filtrated at 0.8 µm and 0.22 µm (Sartorius Stedim Biotech, Aubagne, France).

The filtrate was incubated with an equal volume of an antibiotic solution (2000 U/mL of penicillin/streptomycin and 300 U/mL of neomycin) for 1 h at room temperature. The suspension was then inoculated on the monolayer of the VeroE6 cells, and the flask was incubated at 37 °C for 1 h. After incubation, 5 mL of EMEM with 6% fetal bovine serum (FBS) was added and incubated again at 37 °C for 72 h.

After 72 h, 200 μL of EMEM were collected from each flask for biomolecular testing and the EMEM 6% FBS was replaced after a further 72 h.

At the end of the test a further 200 μL of flask medium was collected for the evaluation of viral load, while the flasks were observed under an inverted microscope Axiovert 25 (Zeiss, Oberkocken, Germany) to evaluate the presence of cytopathic effects [30]. The result was defined on the basis of the cytopathic effect (subjective reading) combined with the results of the RT-PCR test (objective reading) in supernatants [31]. All procedures for viral culture followed laboratory biosafety guidelines and were performed in a biosafety level 3 (BSL-3) laboratory.

### 2.3. RT-PCR

Viral RiboNucleic Acid (RNA) was extracted from the medium of flasks at T0, after 72 h and after 144 h, using the QIAamp Viral RNA Mini Kit, according to the manufacturer’s instructions (Qiagen, Hilden, Germany). Amplification and detection of target genes (N, E, and RdRP) were performed using the commercially available kit GeneFinder COVID-19 Plus RealAmp (Osang Healthcare Co. Ltd., Anyang, Korea) with the CFX96TM instrument (Bio-Rad, Hercules, CA, USA). The cycle threshold (Ct) of each RT-PCR reaction was calculated following the manufacturer’s instructions. The test was considered positive when at least one of the three investigated genes showed a Ct below 40 [30].

## 3. Results

Sixteen patients who died with/from COVID-19 and underwent autopsy between April 2020 and April 2021 were included in this study. As summarized in Table 1, they were split into two groups, the short PMI group, and the long PMI group. Autopsies were conducted according to international guidelines. All patients tested positive for SARS-CoV-2 infection both before death (nasopharyngeal swabs) and during post-mortem investigation (swab performed on lung tissue). During autopsy procedures, a lung specimen was collected and frozen at −80 °C until investigations were performed. The sample was stored in sterile tubes containing later RNA (Cat. 76104, RNA Protect Tissue Reagent, Qiagen). Lung specimens were sent to a Biosafety Level 3 (BSL-3) laboratory for viral culture. We used cultivation from lung tissue swab specimens for this analysis because the lung often shows increased SARS-CoV-2 loads in deceased patients and therefore represents a major infection source during autopsy [32]. The tissues were lyophilized; the supernatant was used for SARS-CoV-2 isolation. The observation lasted for a week. The result was defined based on the cytopathic effect (subjective reading) combined with the positivity of the RT-PCR test (objective reading) in the supernatant. All procedures for viral culture followed laboratory biosafety guidelines.

As summarized in Table 1, even if all patients tested positive at RT-PCR for the SARS-CoV-2 infection before death and at the time of the autopsy. In eight samples (S3, S6, S8, S9, S10, S11, S12, S16), we found differences in RT-PCR positivity between lung swabs and homogenized lung tissues: this discrepancy may be related to the sensitivity of the molecular probes with these samples. Considering the results of cell culture, only one sample (ID = S2) demonstrated virus vitality. The other samples were all negative, demonstrating that the positivity to the swab sample does not demonstrate virus vitality. Contrariwise, it was demonstrated that 24 h after death, in the main site of infection (lung tissue), the virus was inactive and not able to infect.

## 4. Discussion

When a subject die from a respiratory infectious virus, the risk of transmission is related to the virus’ vitality. In the case of SARS-CoV-2, numerous studies have been carried out on post-mortem specimens demonstrating positivity to the molecular test for SARS-CoV-2 [25,33,34], although no studies have been performed to define the time necessary for its inactivation. It is well known that the risk of infection from a cadaver is related to improper handling immediately after death, when pathogens may still be viable. Infection risk decreases in direct relationship to interval time as well as number of viral cells. In a previous study, we demonstrated the possibility to detect the presence of SARS-CoV-2 RNA without evidence of its replication in exhumed patients with a PMI of 78 days [25]. The novelty of this experimental paper is that for the first time it has been demonstrated that there was no viral replication starting from 24 h in samples collected from subjects who had died with/from COVID-19. It should be pointed out that this time range can undoubtedly be lower, considering that only one sample tested positive, in the case of an autopsy performed after 12 h, and with the corpse stored at 0 °C. Several studies have reported the detection of SARS-CoV-2 viral RNA in cadavers; however, it is well known that the detection of viral RNA does not indicate that the body is necessarily infectious. To date, SARS-CoV-2 has been detected in different post-mortem samples, such as swabs sampled on eyes [35], nose, and mouth [36], periodontal tissue [37], respiratory tract [25,38], including nasopharynx, throat, and lungs, and other tissues and body fluids [39,40].

No studies have been demonstrated SARS-CoV-2 transmission from a deceased person to a living subject as well as there are no report concerning infection that have been occurred during both SARS and MERS autopsy or COVID-19 infection [21,41]. This is in contrast with the Ebola disease, where dead bodies are known to be associated with contagion. In a study by Prescott et al., the authors performed research using an animal model (cynomolgus macaques), the viable virus was isolated <7 days post euthanasia; contrariwise, viral RNA was detectable for 10 weeks [42]. It is important to note that the Ebola virus is an RNA virus from the Filoviridae family, while SARS-CoV-2 is an RNA virus from the Coronaviridae family [43,44,45]. In a recent report, the CDC summarized the suggestions in order to manage the corpse of subjects who had died infected by SARS-CoV-2 or Ebola viruses [46]. In Table 2, we summarize the main differences between SARS-CoV-2 and the Ebola viruses.

To date, many autopsies have been performed on subjects who died from/with COVID-19, the lack of reports of a direct link between infection and post-mortem investigation confirmed that autopsy should be considered a safe procedure, particularly when all recommendations are adopted.

The COVID-19 pandemic has increased the number of deaths worldwide; inevitably the risk related to the movement of corpses demands a greater awareness of standard precautions, applying good practice and guidelines [24]. A prudent approach was adopted in consideration of the fact that SARS-CoV-2 was an unknown etiological pathogen. Considering that viral cultures for COVID-19 infectious represent the best way to determine virus viability and infectivity [47], these experimental results suggest that the risks of SARS-CoV-2 transmission may be considered minimal in handling, transporting, and examining deceased persons with COVID-19. Particularly, considering the data provided by this study and the recent literature, it may be confirmed that the risk of cadaveric infection in cases of a person who died from/with COVID-19 is extremely low and related to the first hours after death, becoming very low after 12 h. The results of the present study are also relevant for non-healthcare professionals, such as funeral directors or morticians. To date, there is no scientific evidence of a higher incidence of COVID-19 infection or mortality among these occupational groups, although they were considered as high-risk categories [48,49]. These data suggest that risk reduction measures are being successfully applied [39]. Moreover, as reported in the ad interim guidance of WHO titled “Infection Prevention and Control for the Safe Management of a Dead Body in the Context of COVID-19”, it may be confirmed that cadavers do not transmit the infection at a PMI no less of 12 h [20].

In line with the present results, difficult questions will arise. For example, was a total ban on funerals necessary, or could the “last goodbye” still be said by the families even though there is physical distance?

In European countries, respect for cultural and religious traditions, combined with the dignity of the dead, should always be respected and protected. In a particular and unexpected situation, such as the COVID-19 pandemic, countless difficulties emerged in the management of these delicate aspects, leading, for the first time, families to the painful deprivation of the last goodbye to their loved ones. There has certainly been a lack of scientific evidence, which, by means of targeted studies, would have allowed a rational management of the problem. For example, different governments adopted severe restrictions in body management of people who have died from/with COVID-19, although WHO suggested that the correct use of personal protective equipment (PPE) was enough to guarantee the dignity of the dead, and the respect of cultural and religious traditions [20].

The main limitation of this study is related to the small number of subjects involved in the study. Concerning this consideration, it is important to highlight that the number of subjects who die with/from COVID-19 who underwent autopsy is still low in Italy, although it is usually performed both for clinical and forensic purposes. It is not always possible to obtain a complete set of biological samples to perform the described experimental protocol, testing the subject at different times in order to confirm SARS-CoV-2 infection. This last point represents the strength of this study: in fact, all involved subjects were tested for the SARS-CoV-2 infection in three different moments (before death, immediately after death, and at autopsy); in this way, we have excluded the possibility of false-positives. Another limitation is related to the impossibility to use the same samples at different PMIs because of the small quantity of the collected sample: this consideration could suggest a new study collecting a fresh lung sample during the autopsy (within 12 h) sampling it at different periods (after 24 h, 48 h, etc.) in order to define the time interval necessary to test the viability of the virus, although several important ethical issues should be considered.

## 5. Conclusions

Although the scientific community has produced numerous scientific reports demonstrating the viral RNA detection on a corpse or in body fluids of subjects deceased with/from COVID-19, no studies have been conducted concerning the viability of the virus and the infectivity hazard of the personnel involved to the management of the corpse with suspected, probable or confirmed COVID-19 respiratory infection. This study is the first to attempt to clarify this crucial aspect both for health and non-health workers. Although more than four million people have died from SARS-CoV-2 infection to date, and there is no scientific evidence of a real hazard of infection from a COVID-19 corpse to a live host. It is unclear whether these data are due to the ability of the operators to implement the various indications for the containment of the infectious risk or whether the body turns out to be non-infectious. Based on the obtained data, the infection hazard appears to be very low and limited to the first 12 h after death. However, further studies are needed to confirm the duration of virus viability in various tissues and fluids in various conditions, as well as the likelihood of indirect transmission.

## Figures and Tables

**Table 1:** Samples enrolled for the study with the indication of the relative PMI. All nasopharyngeal and lung swabs tested positive, while only one cell culture (S2) resulted positivity demonstrating virus vitality.

GROUP	ID	Cause of Death	PMI (Time Elapsed from Death to Autopsy)	SARS-CoV-2 Test			
				Nasopharyngeal Swab	Lung Swab	Homogenized Lung Tissue	Cell Culture
**SHORT** **PMI**	S1	interstitial pneumonia with fibrosis	12 h	positive	positive	positive	negative
	S2	multiple organ dysfunction syndrome (MODS)	12 h	positive	positive	positive	positive
	S3	pulmonary edema	24 h	positive	positive	negative	negative
	S4	acute respiratory distress syndrome (ARDS)	24 h	positive	positive	positive	negative
	S5	MODS	48 h	positive	positive	positive	negative
	S6	interstitial pneumonia with fibrosis	48 h	positive	positive	negative	negative
	S7	cardiac failure	72 h	positive	positive	positive	negative
	S8	interstitial pneumonia with fibrosis	72 h	positive	positive	negative	negative
**LONG** **PMI**	S9	ARDS	24 days	positive	positive	negative	negative
	S10	septic shock	32 days	positive	positive	negative	negative
	S11	interstitial pneumonia with fibrosis	45 days	positive	positive	negative	negative
	S12	septic shock	46 days	positive	positive	negative	negative
	S13	interstitial pneumonia with fibrosis	50 days	positive	positive	positive	negative
	S14	ARDS	54 days	positive	positive	positive	negative
	S15	pulmonary edema	61 days	positive	positive	positive	negative
	S16	septic shock	78 days	positive	positive	negative	negative

**Table 2:** The main differences between SARS-CoV-2 and the Ebola virus.

Virus	SARS-CoV-2	Ebola Virus
Category	RNA virus	RNA virus
Family	Coronaviridae	Filoviridae
Origin	Zoonotic	Zoonotic
Transmission	Contact with respiratory droplets of infected subjects. Transmission from asymptomatic individuals.	Contact with blood or body fluid of infected subjects.
Secondary transmission (fomites)	Unknown	Yes
Transmission from deceased subjects	Limited to a few hours after death.	Possible until 7 days after death.

## Data Availability

Data are not publicly available due to protection of personal data and medical confidentiality.
